# Novel biomarker-based model for the prediction of sorafenib response and overall survival in advanced hepatocellular carcinoma: a prospective cohort study

**DOI:** 10.1186/s12885-018-4211-2

**Published:** 2018-03-20

**Authors:** Hwi Young Kim, Dong Hyeon Lee, Jeong-Hoon Lee, Young Youn Cho, Eun Ju Cho, Su Jong Yu, Yoon Jun Kim, Jung-Hwan Yoon

**Affiliations:** 1 Department of Internal Medicine, College of Medicine, Ewha Womans University, Seoul, Republic of Korea; 2 Department of Internal Medicine and Liver Research Institute, Seoul National University College of Medicine, Seoul, Republic of Korea; 3 Department of Internal Medicine, SMG-SNU Boramae Medical Center, Seoul, Republic of Korea; 4 Department of Internal Medicine, Seoul National University Hospital, 101, Daehak-ro, Jongno-gu, Seoul, 03080 Republic of Korea

**Keywords:** Hepatocellular carcinoma, Sorafenib, Response, Biomarker, Prediction

## Abstract

**Background:**

Prediction of the outcome of sorafenib therapy using biomarkers is an unmet clinical need in patients with advanced hepatocellular carcinoma (HCC). The aim was to develop and validate a biomarker-based model for predicting sorafenib response and overall survival (OS).

**Methods:**

This prospective cohort study included 124 consecutive HCC patients (44 with disease control, 80 with progression) with Child-Pugh class A liver function, who received sorafenib. Potential serum biomarkers (namely, hepatocyte growth factor [HGF], fibroblast growth factor [FGF], vascular endothelial growth factor receptor-1, CD117, and angiopoietin-2) were tested. After identifying independent predictors of tumor response, a risk scoring system for predicting OS was developed and 3-fold internal validation was conducted.

**Results:**

A risk scoring system was developed with six covariates: etiology, platelet count, Barcelona Clinic Liver Cancer stage, protein induced by vitamin K absence-II, HGF, and FGF. When patients were stratified into low-risk (score ≤ 5), intermediate-risk (score 6), and high-risk (score ≥ 7) groups, the model provided good discriminant functions on tumor response (concordance [c]-index, 0.884) and 12-month survival (area under the curve [AUC], 0.825). The median OS was 19.0, 11.2, and 6.1 months in the low-, intermediate-, and high-risk group, respectively (*P* < 0.001). In internal validation, the model maintained good discriminant functions on tumor response (c-index, 0.825) and 12-month survival (AUC, 0.803), and good calibration functions (all *P* > 0.05 between expected and observed values).

**Conclusions:**

This new model including serum FGF and HGF showed good performance in predicting the response to sorafenib and survival in patients with advanced HCC.

## Background

Sorafenib is an oral multikinase inhibitor targeting the receptor tyrosine kinase activity of VEGF receptors (VEGFR 1–3), PDGFRb, serine-threonine kinases Raf-1 and B-Raf, c-KIT, and RET [1–3]. Sorafenib therapy prolonged overall survival (OS) with acceptable safety and tolerability in patients with advanced HCC in the SHARP (Sorafenib Hepatocellular Carcinoma Assessment Randomized Protocol) trial and the Asia-Pacific study [4, 5]. Based on these two phase III trials, sorafenib therapy is currently the standard of care for patients with advanced-stage or Barcelona Clinic Liver Cancer (BCLC) stage C HCC and for patients in the control arm of ongoing clinical trials [6, 7]. However, considering the various adverse effects, incomplete response, modest survival benefit and cost of sorafenib therapy, the necessity of predictive biomarkers has been constantly raised, to select patients who could benefit most from this treatment [8].

Cancer biomarkers have been widely used for the prediction of the natural course, prognosis, and treatment response in certain malignancies [9]. Predictive biomarkers can be best explored in the setting of properly designed clinical trials [10]. Baseline plasma angiopoietin 2 and vascular endothelial growth factor (VEGF) were identified as independent predictors of survival in the SHARP study; however, no biomarker was predictive of sorafenibresponse [11]. Because the diagnosis of HCC is usually made without obtaining tumor tissue, serum biomarkers for the prediction of sorafenib response would be of significant benefit for proper selection of patients [12]. Furthermore, there has been no attempt to date to integrate potential serum markers and other relevant clinical characteristics for a response prediction model as an approach toward precision medicine in patients with advanced HCC.

This exploratory study aimed (i) to develop a novel relevant predictive model using a serum biomarker for the prediction of sorafenib response, (ii) to validate this model internally, and (iii) to determine its role in predicting outcome in a prospectively collected database from a large-scale in-hospital cohort.Specifically, we chose analytes for serum biomarker analysis on the basis of the molecular targets (or ligands of those targeted receptors) of sorafenib or those related to the outcome and/or pathogenesis of HCC, including VEGF [11], platelet-derived growth factor receptor β (PDGFRb) [2], CD117 [1], hepatocyte growth factor (HGF) [13], angiopoietin 2 [11], lysyl oxidase-like 2 (LOXL2) [14], and basic fibroblast growth factor (bFGF) [15].

## Methods

### Patients

Between May 2013 and June 2015, a total of 460 consecutive patients who had a diagnosis of HCC and received sorafenib were identified in a prospective HCC cohort registry of Seoul National University Hospital. Written informed consent was obtained from each patient before registration in the cohort. The diagnosis of HCC was based on histological examination or clinico-radiological criteria, with reference to the practice guidelines from the American Association for the Study of Liver Diseases or the European Association for the Study of the Liver [6, 7]. All patients were not indicated for surgical resection, liver transplantation, local ablation, or transarterial chemoembolization at the time of initiation of sorafenib therapy because of their advanced stages at the time of diagnosis or tumor progression despite prior (repeated) locoregional treatments. Among these 460 patients, adequate blood samples were obtained from 161 patients for serum biomarker analyses. Patients with Child-Pugh score ≥ 7 (*n* = 37) were excluded because the outcome assessment of sorafenib therapy in these patients could be misleading, owing to increased risk of mortality from their impaired liver function [16]. Finally, 124 patients were included in the analysis.

The protocol of the present study conformed to the ethical guidelines of the World Medical Association Declaration of Helsinki, and was approved by the Institutional Review Board of Seoul National University Hospital (IRB No. 0506–150-005). All study participants provided written informed consent. REMARK (Reporting Recommendations for Tumor Marker Prognostic Studies) criteria were followed throughout this study [17].

### Treatment scheme and response evaluation

The standard daily oral dose of sorafenib (Nexavar; Bayer HealthCare AG, Leverkusen, Germany) was 800 mg (i.e., 400 mg twice daily) on a continuous dosing schedule. Follow-up evaluation schedules included (i) clinical examination (toxicity assessment and blood tests) 2 weeks after the first administration of sorafenib and every 4 weeks thereafter, and (ii) imaging tests (contrast-enhanced dynamic computed tomography or magnetic resonance imaging) every 6–8 weeks until death or the last follow-up. Dose reduction was allowed in cases of drug-related grade 3 or 4 toxicities. The toxicity grade was assessed before each treatment cycle using the National Cancer Institute Common Toxicity Criteria for Adverse Events (version 4.0). We used three-step dose reduction as follows: 800 mg daily to 600 mg daily, 600 mg daily to 400 mg daily, and 400 mg daily to 400 mg every other day. Treatment response was evaluated every 6–8 weeks, according to the modified Response Evaluation Criteria in Solid Tumors criteria using contrast-enhanced dynamic computed tomography or magnetic resonance imaging [18]. Sorafenib therapy was discontinued if one or more of the following occurred: disease progression, development of intolerable toxicity, or patient refusal.

### Sample collection and biomarker assays

A 5-mL blood sample was collected from each study participant at baseline (before the initiation of sorafenib therapy), and was centrifuged at 1500 rpm for 15 min to separate the serum. The serum samples were then stored at ≤ − 70 °C in 1.5-mL aliquots until further assays.

The serum concentrations of biomarkers were measured with commercially available ELISA kits for angiopoietin 2 (R&D Systems, Minneapolis, MN, USA), VEGF (eBioscience, San Diego, CA, USA), PDGFRb (Raybiotech, Norcross, GA, USA), HGF (R&D Systems), CD117 (R&D Systems), LOXL2 (USCN Life Science, Wuhan, China), and bFGF (R&D Systems).

### Statistical analysis

For baseline characteristics, continuous variables are expressed as medians and ranges, and categorical variables as frequencies with percentages. Survival analysis was performed using the Kaplan-Meier method from the date of initial diagnosis of HCC to the date of death or last follow-up, with the log-rank test to compare subgroups. Single binary logistic regression analysis was used to identify relevant features associated with response to sorafenib, in which variables with *P* < 0.1 were subsequently included in the multivariate analysis. The selected variables for logistic regression analysis included clinical characteristics, laboratory parameters, serum biomarkers, and hepatic fibrosis indices [19–22]. Forward and backward stepwise selection procedures were sequentially used to select the best-fitted model on the basis of the Akaike information criterion [23]. In the final model, scores (0, 1, 2) were assigned to the corresponding levels of categorical covariates. For continuous covariates, scores were assigned to the corresponding subranges within cutoff values, to maximize the concordance index (c-index). Hence, the risk score for the prediction of sorafenib response was calculated in each patient through the summation of the scores of the covariates in the final prediction model. Patients were further categorized into subgroups according to their predictive scores. For an internal validation of the predictive model, a 3-fold cross-validation was performed. Calibration function was examined by comparing the observed response with the expected response estimated with the risk score, using the Hosmer-Lemeshow test. In addition, survival prediction was performed among subgroups according to the risk scores, with internal validation and examination of calibration function, in the same manner as that for the response prediction.

All tests were based on a two-sided probability, and *P* < 0.05 was considered statistically significant. All statistical analyses were performed with R language ver. 3.1.1 (R Foundation for Statistical Computing, Vienna, Austria).

## Results

### Patient characteristics and treatment outcomes

Table 1 summarizes the baseline characteristics of the enrolled patients according to their best objective responses to sorafenib, i.e., progressive disease (PD) vs. non-PD. During the treatment period (median, 2.6 months; interquartile range, 1.4–3.8 months), 44 patients had no disease progression (non-PD group: complete response, 2 patients; partial response, 10 patients; and stable disease, 32 patients) and 80 patients had disease progression (PD group). The median time to progression was 2.7 months (95% confidence interval [CI], 2.4–3.1). The main underlying cause of liver disease was hepatitis B infection (59.1% in the non-PD group, 81.2% in the PD group). There was no significant difference in the frequencies of macrovascular invasion or extrahepatic spread and baseline Child-Pugh score between the two groups. Platelet count was significantly lower in the PD group than in the non-PD group (*P* = 0.006). Of the baseline tumor markers, PIVKA-II (protein induced by vitamin K absence-II) was significantly higher in the PD group than in the non-PD group (*P* = 0.003), unlike alpha-fetoprotein (*P* = 0.187). Among the serum biomarkers, only bFGF was significantly higher in the PD group than in the non-PD group (*P* < 0.001).

**Table 1:** Baseline characteristics according to the sorafenib response

Variable	All (*n* = 124)	Non-PD (*n* = 44)	PD (*n* = 80)	*P*
Age (year)	61 ± 11	62 ± 10	61 ± 12	0.707
Sex, N (%)	107 (86.3)	36 (81.8)	71 (88.8)	0.423
ECOG performance status 0, N (%)	112 (90.3)	42 (95.5)	70 (87.5)	0.210
Etiology, N (%)				0.02
HBV	91 (73.4)	26 (59.1)	65 (81.2)	
HCV	21 (16.9)	10 (22.7)	11 (13.8)	
Alcohol	12 (9.7)	8 (18.2)	4 (5)	
Body mass index (kg/m^2^)	22.3 ± 3.1	23.1 ± 3.2	21.9 ± 3.0	0.052
Macrovascular invasion, N (%)	39 (31.5)	11 (25.0)	28 (35.0)	0.344
Extrahepatic spread, N (%)	53 (42.7%)	16 (36.4)	37 (46.2)	0.382
BCLC stage C, N (%)	83 (66.9%)	25 (56.8)	58 (72.5)	0.185
Child-Pugh score, N (%)				0.492
5	114 (91.9)	42 (95.5)	72 (90)	
6	10 (8.1)	2 (4.5)	8 (10)	
MELD	8.8 ± 2.2	8.36 ± 1.54	8.96 ± 2.47	0.1
Albumin (g/dL)	3.79 ± 0.42	3.78 ± 0.46	3.81 ± 0.41	0.739
Total bilirubin (mg/dL)	1.06 ± 0.50	0.99 ± 0.47	1.09 ± 0.51	0.273
Prothrombin time (INR)	1.14 ± 0.10	1.13 ± 0.09	1.15 ± 0.1	0.299
Creatinine (mg/dL)	0.89 ± 0.56	0.87 ± 0.13	0.9 ± 0.69	0.705
Platelet (×10^3^/μL)	122 (17–787)	113 (17–291)	128 (52–787)	0.006
Alpha-fetoprotein (ng/mL)	128.8 (1.3–730,000)	114.7 (2.6–226,100)	152.2 (1.3–730,000)	0.187
PIVKA-II (mAU/mL)	109.5 (12–339,858)	25.5 (12–25,884)	351 (15–339,858)	0.003
Angiopoietin 2 (ng/mL)	2962.7 (1099.4–12,907.8)	2762.2 (1289.6–8360.4)	3026.9 (1099.4–12,907.8)	0.109
CD117 (ng/mL)	3.76 (1.01–226.85)	3.36 (1.01–10.23)	3.87 (1.10–226.85)	0.234
bFGF (pg/mL)	2.38 (0–61.57)	1.65 (0–14.62)	3.07 (0.43–61.57)	< 0.001
HGF (pg/mL)	1781.9 (913.9–6116.0)	1605.7 (913.9–4152.4)	1965.2 (1020.3–6117.0)	0.056
LOXL2 (ng/mL)	0.816 (0.034–4.007)	0.718 (0.034–4.007)	0.850 (0.039–3.765)	0.792
PDGFRb (pg/mL)	2045.6 (647.5–22,904.5)	2087.9 (777.7–22,904.5)	1965.3 (647.5–8214.9)	0.419
sVEGFR1 (ng/mL)	0.16 (0.04–13.65)	0.16 (0.06–13.65)	0.14 (0.04–0.53)	0.314
AAR	1.29 (0.58–11.6)	1.31 (0.58–2.67)	1.29 (0.61–11.6)	0.105
APRI	1.01 (0.17–6.54)	1.17 (0.17–4.54)	0.95 (0.17–6.54)	0.847
FIB-4	4.0 (0.6–21.0)	4.4 (0.9–21.0)	3.6 (0.6–12.3)	0.161
Lok index	0.66 ± 0.24	0.67 ± 0.24	0.65 ± 0.24	0.68
P2/MS	29.6 (0.46–1060.7)	27.3 (0.5–202.4)	36.7 (6.2–1060.7)	0.102

Values are expressed as mean ± standard deviation, medians (ranges), or frequencies (percentages)

*Abbreviations: PD*, progressive disease, *ECOG* Eastern Cooperative Oncology Group, *HBV* hepatitis B virus, *HCV* hepatitis C virus, *BCLC* Barcelona Clinic Liver Cancer, *MELD* model for end-stage liver disease, *INR* international normalized ratio, *PIVKA-II* protein induced by vitamin K absence-II, *Ang2* angiopoietin-2, *bFGF* basic fibroblast growth factor, *HGF* hepatocyte growth factor, *LOXL2* lysyl oxidase-like 2, *PDGFRb* platelet-derived growth factor receptor β, *sVEGFR1* soluble vascular endothelial growth factor receptor-1, *AAR* aspartate aminotransferase/alanine aminotransferase ratio, *APRI* aspartate aminotransferase to platelet ratio index, *FIB-4* fibrosis 4

The median follow-up duration was 6.6 months (interquartile range, 4.3–12.1 months) and OS was 11.2 months (95% CI, 9.4–13.7).

### Development of a scoring system for the prediction of sorafenib response

**Fig. 1:**
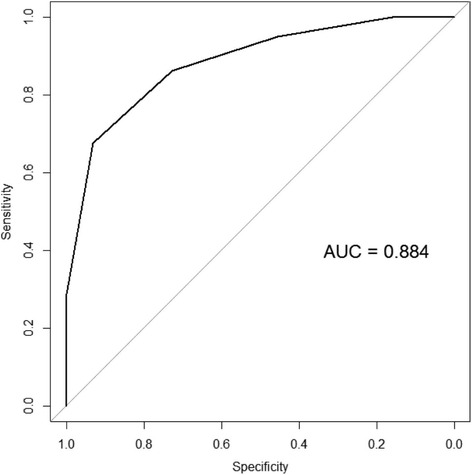
Area under the receiver-operating curve (AUC) analysis for exploring a threshold score for predicting sorafenib response. The risk score was calculated by using six covariates from the multivariable binary logistic regression analysis (Table 3). When the score was 6 or higher, the sorafenib response was expected to be progressive disease with maximal sensitivity and specificity

From the single binary logistic regression analysis, variables with *P* < 0.1 were first selected for multivariate analysis, including etiology, platelet count, PIVKA-II, bFGF, macrovascular invasion, extrahepatic spread, BCLC stage, and log HGF (Table 2). Among these variables, multivariate logistic regression analysis with forward stepwise selection process identified six variables for the final model (Table 2), which included four tumor-related factors (BCLC stage, bFGF, log PIVKA-II, and log HGF) and two liver disease-related factors (etiology and platelets). The risk scoring system for the prediction of sorafenib response was generated using these six covariates, in which a score of 0, 1, or 2 was given to each cutoff value of the six covariates (Table 3). The risk score for each patient was calculated through the summation of the scores of the six covariates, and the scores ranged from 0 to 12. Sorafenib response was expected to be PD if the risk score was > 6, showing maximal sensitivity and specificity with this cutoff level (Fig. 1). When all patients were stratified into the low-risk group (risk score ≤ 5; *n* = 43), intermediate-risk group (risk score 6, *n* = 24), and high-risk group (risk score ≥ 7, *n* = 57), the model provided good discriminant functions on sorafenib response (c-index, 0.884; 95% CI, 0.827–0.941). In the internal validation with 3-fold cross-validation, the model maintained good discriminant functions on tumor response (c-index, 0.825; 95% CI, 0.745–0.906), and the Hosmer-Lemeshow *P*-value for the calibration function of the risk score was 0.945.

**Table 2:** Binary logistic regression analysis for sorafenib response

Variable		Univariate analysis		Multivariate analysis	
		OR (95% CI)	*P*	OR (95% CI)	*P*
Age (year)		0.994 (0.963–1.027)	0.719		
Sex	(Female)	0.570 (0.203–1.603)	0.287		
Body mass index (kg/m^2^)		0.885 (0.783–1.000)	0.050		
ECOG PS	(1 vs. 0)	3.000 (0.627–14.358)	0.169		
Etiology	HCV vs. nonviral	0.440 (0.167–1.16)	0.097	0.612 (0.179–2.088)	0.433
	HBV vs. nonviral	0.200 (0.055–0.722)	0.014	0.133 (0.027–0.666)	0.014
Ascites	2 vs. 1	2.333 (0.473–11.506)	0.298		
Platelet (×10^3^/μL)		1.006 (1.001–1.011)	0.017	1.005 (0.999–1.011)	0.103
Creatinine (mg/dL)		1.115 (0.529–2.348)	0.775		
Albumin (g/dL)		1.168 (0.488–2.794)	0.727		
Total bilirubin (mg/dL)		1.541 (0.698–3.405)	0.285		
Prothrombin time (INR)		6.922 (0.164–291.871)	0.311		
MELD		1.208 (0.937–1.558)	0.145		
Macrovascular invasion		1.615 (0.71–3.677)	0.253		
Extrahepatic spread		1.506 (0.708–3.205)	0.288		
BCLC stage	B vs. A	1.000 (0.315–3.174)	1.000	0.808 (0.203–3.224)	0.763
	C vs. A	2.895 (1.002–8.364)	0.050	3.476 (0.987–12.243)	0.052
Log AFP (ng/dL)		1.013 (0.779–1.317)	0.925		
Log PIVKA-II (mAU/mL)		1.989 (1.359–2.911)	< 0.001	1.724 (1.124–2.644)	0.013
Log Angiopoietin 2 (ng/mL)		3.754 (0.534–26.374)	0.183		
Log CD117 (ng/mL)		2.895 (0.612–13.687)	0.18		
bFGF (pg/mL)		1.321 (1.092–1.598)	0.004	1.326 (1.077–1.634)	0.008
Log HGF (pg/mL)		16.534 (1.275–214.349)	0.032	23.438 (0.750–732.109)	0.072
LOXL2 (ng/mL)		0.769 (0.533–1.11)	0.160		
Log sVEGFR1 (ng/mL)		0.272 (0.047–1.592)	0.149		
AAR		1.373 (0.84–2.246)	0.206		
APRI		0.971 (0.718–1.314)	0.85		
FIB4		0.926 (0.842–1.02)	0.12		
Lok index		0.722 (0.154–3.386)	0.68		
P2/MS		1.004 (0.998–1.01)	0.179		

*Abbreviations: OR* odds ratio, *CI* confidence interval, *ECOG PS* Eastern Cooperative Oncology Group performance status, *HBV* hepatitis B virus, *HCV* hepatitis C virus, *BCLC* Barcelona Clinic Liver Cancer, *MELD* model for end-stage liver disease, *INR* international normalized ratio, *AFP* alpha-fetoprotein, *PIVKA-II* protein induced by vitamin K absence-II, *Ang2* angiopoietin-2, *bFGF* basic fibroblast growth factor, *HGF* hepatocyte growth factor, *LOXL2* lysyl oxidase-like 2, *PDGFRb* platelet-derived growth factor receptor β, *sVEGFR1* soluble vascular endothelial growth factor receptor-1, *AAR* aspartate aminotransferase/alanine aminotransferase ratio, *APRI* aspartate aminotransferase to platelet ratio index, *FIB-4* fibrosis 4

**Table 3:** Scoring system for the prediction of sorafenib response by using six covariates selected in the multivariable binary logistic regression analysis

Variable	Cutoff value	Score
bFGF (pg/mL)	< 2.0	0
	2–5.5	1
	≥5.5	2
Log PIVKA-II (mAU/mL)	< 30	0
	30–1780	1
	≥1780	2
BCLC stage	A, B	0
	C	1
Etiology	Nonviral	0
	HCV	1
	HBV	2
Log HGF (pg/mL)	< 1380	0
	1380–1860	1
	≥1860	2
Platelet (×10^3^/μL)	< 70	0
	70–184	1
	≥184	2

*Abbreviations: bFGF* basic fibroblast growth factor, *PIVKA-II* protein induced by vitamin K absence-II, *BCLC* Barcelona Clinic Liver Cancer, *HGF* hepatocyte growth factor

### Effect of the sorafenib response score on the OS

**Fig. 2:**
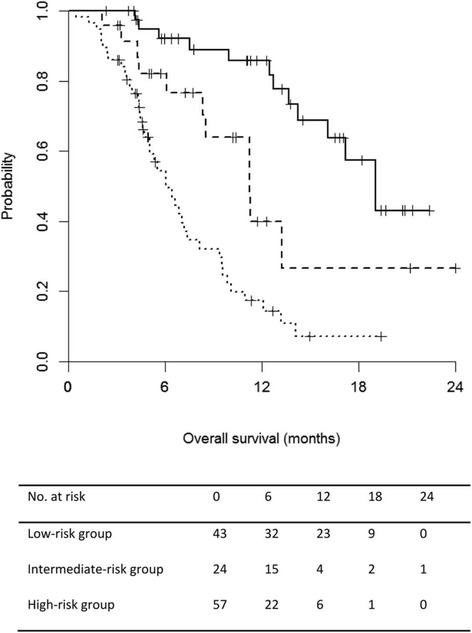
Kaplan-Meier survival curves for subgroups according to the risk scores. The median overall survivals were 19.0 months in group A (score ≤ 5, solid black line), 11.2 months in group B (score 6, dashed line), and 6.1 months in group C (score ≥ 7, dotted line) (*P* < 0.001, log-rank test)

Median OS duration was 19.0 months in the low-risk group (95% CI, 16.1–not available [N/A]), 11.2 months in the intermediate-risk group (95% CI, 8.5–N/A), and 6.1 months in the high-risk group (95% CI, 5.0–8.1) (*P* < 0.001, Fig. 2). Univariate Cox proportional hazards analysis with the above-mentioned risk scoring system showed a significant difference between group A and group C (*P* < 0.001, Table 4). The area under the receiver-operating curve for OS prediction was 0.825 (95% CI, 0.734–0.915) at 12 months. In the internal validation with 3-fold cross-validation, the area under the receiver-operating curve for OS prediction at 12 months was 0.803 (95% CI, 0.699–0.907). The Hosmer-Lemeshow *P*-value for the calibration function of the risk score was 0.207.

**Table 4:** Cox proportional hazards analysis for predicting survival in the subgroups according to risk scores

Risk group	HR (95% CI)	*P*
Intermediate risk vs. low risk	2.529 (1.120–5.707)	0.025
High risk vs. low risk	6.577 (3.419–12.655)	< 0.001

Risk groups according to risk scores: low-risk group, score ≤ 5; intermediate-risk group, score 6; high-risk group C, score ≥ 7

*Abbreviations: HR* hazard ratio, *CI* confidence interval

## Discussion

We developed a new prediction model for sorafenib response that combines relevant serum markers, tumor-related factors, and cirrhosis-related factors in a scoring system. The risk score showed good performance in predicting the response to sorafenib and survival in patients with advanced HCC in our cohort. The robustness of the prediction model was also verified with internal validation.

Since the approval of sorafenib in 2007, no clinical trial with newer agents has shown superior outcomes as a first-line treatment, until recently [8]. More importantly, there is no predictive biomarker for the selection of patients who could benefit most from sorafenib, unlike other malignancies including breast cancer, lung cancer, and melanoma [24–26]. Most of the prospective clinical trials of molecular targeted therapies in patients with HCC to date were not designed for a prespecified patient population based on molecular classification and biomarkers. In addition, because noninvasive diagnosis is feasible in most cases with the characteristic imaging features, this omission of obtaining tumor tissue is another obstacle for biomarker exploration in HCC [12]. Thus, we explored candidate biomarkers for the prediction of sorafenib response in a prospectively collected clinical database and serum samples from an in-hospital cohort.

Comparing the present study with the Asia-Pacific sorafenib study [5], the median time to progression (2.7 months) was similar; however, the median OS (11.2 months) was longer in the present study than in the Asia-Pacific study (6.5 months). Although most of the enrolled patients had chronic hepatitis B in both studies, patients in the Asia-Pacific study had more advanced tumors in that macrovascular invasion or extrahepatic spread was more frequently observed, which might have been responsible for the difference in OS. Indeed, a subanalysis of the GIDEON study from Japan reported that the median OS duration of patients with sorafenib therapy was 17.4 months in those with Child-Pugh class A disease and 4.9 months in those with Child-Pugh class B [27]. The longer OS in Japanese patients with Child-Pugh class A might have resulted from the inclusion of fewer patients in BCLC stage C, compared with our study population (54.7 vs. 66.9%).

The selected covariates for the prediction of sorafenib response from the logistic regression analysis included etiology (B-viral), platelet count, BCLC stage, PIVKA-II, serum bFGF, and serum HGF. Concerning the etiology, hepatitis B-associated HCC was suggested as one of the possible reasons for the shorter OS in the Asia-Pacific study than that in the SHARP study [5]. High serum PIVKA-II level has been reported as a significant predisposing factor for aggressive HCC biology including vascular invasion, intrahepatic spread, and extrahepatic spread [28–30]. Among serum angiogenic factors, bFGF and HGF were selected as covariates in the final risk scoring model. FGF2 or bFGF is known to stimulate HCC proliferation through an autocrine mechanism, to activate HCC invasion, and to induce angiogenesis [15]. In addition, previous studies reported that bFGF level increased as the tumor stage became more advanced, and was predictive of worse postoperative survival in patients with HCC [31, 32]. HGF/MET (mesenchymal-epithelial transition) factor is frequently dysregulated, playing a pivotal role in malignancies, including HCC [33]. Activation of the c-MET pathway promotes tumor cell growth, angiogenesis, and metastasis, leading to more aggressive forms of HCC and poor outcomes [34]. A scoring system was developed by combining all the above-mentioned covariates, which showed good predictive performance for sorafenib response (area under the receiver-operating curve of 0.884 at the cutoff score of 6). More desirably, when patients were stratified into three subgroups according to risk scores, OS of the high-risk group was significantly shorter than that of the low-risk group. Recent clinical trials have investigated the efficacy of newer molecular targeted agents in advanced HCC, such as tivantinib (NCT01755767) and lenvatinib (NCT01761266). Tivantinib is an HGF/c-MET inhibitor, and lenvatinib is a multitargeted receptor kinase inhibitor against VEGFR 1, 2, and 3; fibroblast growth factor receptor 1, 2, 3, and 4; platelet-derived growth factor receptor; RET; and c-KIT [33, 35]. Given that the signaling pathways of these new agents do not entirely overlap with the targets of sorafenib, the relevance of HGF and bFGF in the prediction of the response to and the outcome of sorafenib therapy suggests the possibility of an individualized approach in the selection of systemic agents in this difficult-to-treat population, based on the prediction model in the present study. Lastly, platelets are the source of multiple growth factors and cytokines, and are known to promote tumor growth, angiogenesis, and metastatic potential [36–38]. Several reports have provided evidence supporting the role of platelets in HCC, e.g., reduction of hepatitis B virus–associated experimental HCC by platelet inhibitors [39], antagonism of sorafenib action by platelet factors in HCC cell lines [40], and complete remission of advanced HCC with sorafenib in combination with clopidogrel [41]. Taken together, platelet count was also included in the final risk prediction model in the present study; however, its precise role in molecular targeted therapy needs more investigation.

The results and the risk scoring system of our study need to be interpreted and applied with caution owing to the following limitations. Firstly and most importantly, although the samples for various biomarker assays were archived in a prospective cohort, multiple use of statistical algorithms raises concerns about false-positive results or overfitting [42, 43]. The most desirable setting to avoid these concerns would be a prospective trial that focuses on a specific (group of) biomarker(s) [42]. However, exploration of predictive biomarkers for sorafenib response in a new trial is not logistically feasible, except for large-scale comparative studies with newer agents [44]. Thus, we chose a retrospective analysis as an alternative approach using prospectively collected samples. Secondly, a split-sample method for the validation of the results could not be applied in our cohort owing to the limited number of study subjects [45]. Instead, we performed internal validation using 3-fold cross-validation. An external validation with consistent results is a prerequisite for the application of this scoring system in other patient populations with a larger sample size [42]. Finally, differences in the baseline clinical characteristics of the study subjects also hinder the imprudent generalization of our results in patient populations with a different etiology or tumor status.

## Conclusions

In conclusion, we developed a biomarker-based prediction model of sorafenib response and survival from a prospective cohort. By using baseline serum bFGF and HGF levels as covariates, a total score of < 6 could be a relevant cutoff value for selecting patients who are most likely to benefit from sorafenib therapy. Furthermore, the cutoff value may also be used as guide to avoid unnecessary toxicity and inconvenience in patients with a score above this cutoff.

## Abbreviations

AUR: Area under the curve
BCLC: Barcelona Clinic Liver Cancer
bFGF: Basic fibroblast growth factor
C-index: Concordance-index
FGF: Fibroblast growth factor
HCC: Hepatocellular carcinoma
HGF: Hepatocyte growth factor
LOXL2: Lysyl oxidase-like 2
N/A: Not available
OS: Overall survival
PD: Progressive disease
PDGFRb: Platelet-derived growth factor receptor β
PIVKA: Protein induced by vitamin K absence-II
REMARK: Reporting Recommendations for Tumor Marker Prognostic Studies
SHARP: Sorafenib Hepatocelluar Carcinoma Assessment Randomized Protocol
VEGF: Vascular endothelial growth factor
VEGFR: Vascular endothelial growth factor receptor
